# Breastfeeding Support Rooms and Their Contribution to Sustainable Development Goals: A Qualitative Study

**DOI:** 10.3389/fpubh.2021.732061

**Published:** 2021-12-23

**Authors:** Carolina Belomo De Souza, Sonia Isoyama Venancio, Regina Paula Guimarães Vieira Cavalcante da Silva

**Affiliations:** ^1^Department of Pediatrics, Federal University of Paraná, Curitiba, Brazil; ^2^Health Institute, Secretariat of Health of São Paulo, São Paulo, Brazil

**Keywords:** breastfeeding, breastfeeding support, breastfeeding promotion, breastfeeding policies, lactation workplace programs, program evaluation, sustainable development, sustainable development goals

## Abstract

**Objectives:** Breastfeeding support rooms are low-cost interventions that may prolong breastfeeding and improve work performance. Thus, we sought to understand the experiences and perceptions of working women who use breastfeeding support rooms and the potential contribution to sustainable development goals.

**Methods:** Descriptive and exploratory research was conducted through convenience sampling of women working in companies with breastfeeding support rooms in the state of Paraná, Brazil. A semi-structured questionnaire was applied through interviews and online self-completion.

**Results:** Fifty-three women between 28 and 41 years old participated in the study. In addition, 88.7% had graduated from college, and 96% were married. From the women's experiences and perceptions, we identified that breastfeeding support rooms contribute to prolonged breastfeeding, improve physical and emotional well-being, allow women to exercise their professional activities comfortably, contribute to women's professional appreciation for the excellent relationship between employees and employers.

**Conclusion:** In this novel study, we demonstrate how, from a female point of view, breastfeeding support rooms can contribute to 8 of the 17 sustainable development goals and should therefore be encouraged and promoted.

## Introduction

Raising breastfeeding indicators is an international priority (1). The proven benefits to maternal and child health are indisputable, as the practice of breastfeeding affects the lives and health of women and children and contributes to the development of human capital (2, 3). Moreover, the benefits extend to low, middle, and high-income countries (3).

The relationship between the promotion, protection, and support of breastfeeding and its contribution to the achievement of several Sustainable Development Goals (SDG) is already established, although breastfeeding is not explicitly embedded in the SDG (2, 3). The SDG corresponds to 17 global goals defined in 2015 at the United Nations General Assembly, under A/RES/70/1: Transforming our World: the 2030 Agenda for Sustainable Development (4, 5).

Lack of breastfeeding support, especially in the workplace, discourages women from maintaining breastfeeding (3, 6). Women have been increasingly joining the workforce and the period after the return from maternity leave represents one of the main challenges for continued breastfeeding (7, 8).

International measures to support breastfeeding in the workplace advise that countries respect the minimum standards set by the International Labor Organization (ILO) in conventions No. 183 and No. 191 to ensure maternity and work protection, including requiring maternity leave of at least 14 weeks and breastfeeding breaks during the workday (9).

In Brazil, the Working Women who Breastfeed (*Mulher Trabalhadora que Amamenta–*MTA) action was created in 2010 by the Ministry of Health to bring together strategies to support breastfeeding at work. Later, in 2015, MTA was added to the Breastfeeding and Complementary Foods axis of the National Policy on Integral Attention to the Child's Health (Pnaisc) (10). MTA has three axes: (1) incentive to the optional extension of maternity leave, which is currently 120 days, to 180 days, (2) the right to daycare or a daycare voucher, which is mandatory for all companies with more than 30 women above the age of 16, and (3) the right to two breastfeeding breaks, which are mandatory for all companies until the child is 6 months old, and the incentive to the implement breastfeeding support rooms (BSRs), which is optional for all companies.

The BSRs and breaks to breastfeed or pump breast milk during the workday are low-cost measures that can reduce absenteeism, improve employee performance, commitment, and retention, and reduce barriers for employees who breastfeed (3).

In addition, BSRs and breastfeeding breaks in the workplace can increase a woman's intention to continue breastfeeding after maternity leave (11) and may increase the chances of breastfeeding for 6 months by 25% (3, 12). As a result, BSRs lead to an increase in the rates of both exclusive and continued breastfeeding (13–16) and can contribute to achieving the global goals set by the World Health Organization and United Nations Children's Fund for 2030, which establish the global target of exclusive breastfeeding for 70% of infants <6 months of age, 80% of infants <12 months old, and 60% of infants under 2 years old (1).

To the best of our knowledge, there are no studies investigating BSRs from a user's point of view, nor how BSRs contribute to achieving goals established by the SDG for 2030. Thus, this study aims to understand the perceptions and experiences of female employees who use BSRs and relate these perceptions to the potential achievement of the international goals set for 2030 to raise breastfeeding rates and achieve the SDG. This study is part of doctoral research that evaluated BSRs in southern Brazil.

## Methods

### Design

This is exploratory, descriptive, and qualitative research. It was conducted between December 2019 and December 2020 but interrupted for 3 months and adjusted due to the global COVID-19 pandemic.

### Sampling

The study selected participants *via* convenience sampling, usual for pilot studies, from a population of 130 female employees who used BSRs after returning to work. In addition, we selected female employees from eight companies in the state of Paraná, in southern Brazil, one of the most developed regions of the country, that implemented BSRs and whose BSR was certified by the Ministry of Health until December 2017, which correspond to 88.9% of companies with active BSR in the state.

All women on maternity leave in the year before the beginning of the study and who used the BSR in their companies were eligible for the study. However, we excluded female employees from companies whose BSR had not been used in the 6 months before the start of the study.

### Data Collection

The data were collected using a semi-structured questionnaire divided into two parts: (1) the profile of the women who used the BSR: time of employment at the company, position held, weekly working hours; marital status (married, common law, single, divorced, or widowed), education (incomplete/complete primary education, incomplete/complete secondary education, incomplete/complete undergraduate, or graduate education), age, and whether they had a 180-day maternity leave (yes/no), and (2) open questions about their experiences with BSRs: what it was like to use them and their opinion of BSRs in the companies, as seen in Table 1.

**Table 1:** Information collected from women who used Breastfeeding Support Rooms (*N* = 53) Paraná, Brazil, 2019–2020.

**Collected information**	**Questions**
Profile of women who used BSRs	• How old are you? • What is your marital status? (married, common law marriage, single, divorced, or widowed) • What is your highest level of education? (incomplete/complete primary education; incomplete/complete secondary education; incomplete/complete undergraduate or graduate education) • How long have you worked for this company? • What position do you currently hold at the company? • How much time do you work per week? • Did you have 180 days of maternity leave? (Yes or No)
Experiences and opinion about BSRs	• How was your experience using your company's breastfeeding support room? • What is your opinion about the breastfeeding support room in companies?

The study began with the application of the questionnaire through in-person interviews conducted within company BSRs. However, at the beginning of the data collection stage, due to the COVID-19 pandemic, we decided to interrupt the research and reflect on alternative approaches. Therefore, we requested the Ethics Committee to include data collected through online self-completion questionnaires (Google Forms), including a consent form. The companies participating in the study sent invitations to their female employees, both for the interviews, announcing the date and time that the researcher (first author) would be conducting the interviews and the online self-completion questionnaire submitted within 60 days. The in-person interviews were audio-recorded and transcribed word-for-word. The answers obtained through the online questionnaire were used precisely as they were recorded in the answer sheet.

### Data Analysis

The Statistic 10.0 (StatsoftR) program was used for the sample description, and the variables studied were expressed in absolute and relative frequencies.

Qualitative analysis was carried out by the thematic categorical content analysis, following three steps (17): (1) material organization, (2) coding, and (3) result interpretation. From the categories or themes found, we deductively sought to identify their relationship with the SDG. The data were analyzed using the Atlas.ti version 9.0 software (18), which contributed to the data management.

For this study, the criteria proposed by the Journal Article Reporting Standards [American Psychological Association (19)] for qualitative research were respected.

## Results

### Sample Characteristics

The study included 53 women, 40.8% of BSR users in Paraná. Of these, 8 (15%) female workers participated by semi-structured interview, and 45 (85%) answered an online self-completion questionnaire.

All-female employees included in this study were between 28 and 41 years old. Thirty (72%) women were between 31 and 40 years old, 47 (88.7%) had graduated college, 51 (96%) were married or living in a common-law marriage. Eleven (21%) women have worked for their company from 1 to 5 years and 42 (79%) for 6 years or more. Most of the participants, 43 (81%), had a 40–44 h weekly workday. The types of positions held by the female employees are available in Table 2. In addition, all women (100%) had 180 days of maternity leave.

**Table 2:** Positions occupied by women who used breastfeeding support rooms (*n* = 53), Brazil, 2019–2020.

**Categories-*N* (%)**	**Types of positions**	***N* (%)**
Administrative-24 (45.2%)	Assistants	2 (3.77%)
	Technicians	6 (11.32%)
	Analysts	12 (22.64%)
	Consultants	2 (3.77%)
	Specialists	2 (3.77%)
Management-6 (11.3%)	Managers	4 (7.54%)
	Coordinators	2 (3.77%)
Health-related-5 (9.5%)	Nurses	3 (5.66%)
	Nutritionists	1 (1.88%)
	Biologists	1 (1.88%)
Customer service-8 (15%)	Call center operators	8 (15.09%)
Research-1 (2%)	Researchers	1 (1.88%)
Operations-9 (17%)	Engineers	4 (7.54%)
	Logistics Planners	1 (1.88%)
	Assemblers	1 (1.88%)
	Laboratory and civil engineering technicians	2 (3.77%)
	Technologists	1 (1.88%)

### Experiences With BSR Use

We consider that all the experiences with BSRs were positive. From the participating reports, we identified that the experience categories are related to the fact that the BSRs: (1) allows for breastfeeding continuity, (2) contributes to the bonding and well-being of the child, (3) provides comfort and emotional well-being for the woman, (4) provides comfort and physical well-being for the woman, (5) is related to the adequate, quiet place with all the necessary infrastructure for breastfeeding, and (6) allows women to donate breast milk to a milk bank, as seen in Table 3.

**Table 3:** Experiences with breastfeeding support rooms among women (*n* = 53), Brazil, 2019–2020.

**Categories**	**Quotes**
**Positive points**	
Allows continuity of breastfeeding	“[…] it enabled me to continue breastfeeding in my return to work at such a delicate and special moment in my life. […]” (M3); “[…] helped me maintain breastfeeding without having to introduce formula.” (M9); “Great experience. I used up my milk at lunchtime, so I was able to guarantee my babies would drink breast milk for a longer period” (M25); “The room enabled me to continue breastfeeding, and I did not have to introduce formulas to them. […]. Essential to maintain milk production and ensure milk when I was absent” (M29); “Very important to continue breastfeeding my daughter […]” (M41); “Very positive, because of it, I can allow my child to exclusively breastfeeding” (M47);
Contributes to the mother-child bond and well-being	“It was extremely important to my son's well-being […]” (M1); “It was extremely important to extend the mother and child bond” (M4);
Provides comfort and emotional well-being for the woman	“I felt welcomed by having a space prepared […]” (M3); “Very rewarding, my daughter did not consume any other milk/formula, only breast milk. […]” (M17); “Wonderful, I felt safe, happy […]” (M20); “I felt respected because after returning to my job, I was able to pump and feed it to the baby later and for the comfort during the working time” (M21); “It was wonderful, I felt welcomed, safe, cared for. Very calm” (M23); “Wonderful. It brought me peace to be able to continue breastfeeding my son. […]” (M27);
Comfort and physical well-being for women	“It was a relief. When my first daughter was born, I suffered so much, physically and psychologically, because I had recurrent mastitis and had no place or time to pump for relief. It was depressing. This time the much-requested room was available […].” (M8); “[…] A major benefit was being able to pump milk and avoid mastitis, which happened when I had my first daughter, precisely because I had no way to pump milk.” (M15); “Comfortably pumping milk and having a place to store it was fundamental to keep breastfeeding painlessly” (M18); “[…] I was relieved of swollen breast pain, especially by 4 pm.” (M27); “[…] The room was essential. […] Also, to relieve any discomfort throughout the day.” (M29); “Great! I overproduced milk, so I needed to pump, and having a proper environment made all the difference.” (M40);
Related to the proper quiet location and with all the necessary infrastructure	“[…] It is evident that every detail in this space has been thought out with great care. I am so thankful for it.” (M3); “ Wonderful. I had a quiet and private place where I could pump milk […].” (M9); “I had everything I needed. I wish I had used it longer.” (M14); “It was a very good, quiet, appropriate location. Good to have a place to store. […]” (M15); “[…] it was a comfortable and safe place where I could pump and freeze milk for my child every day. […]” (M41); “[…]. The option to have a suitable and private place, quiet, makes the moment more appropriate to pump milk.” (M51);
Allowed donation to the Human Milk Bank	“[…] I even managed to donate to the milk bank for over a year.” (M8); “[…]. For some time, I was able to donate breast milk” (M17); “[…] besides easing the process of donation to the milk bank” (M16); “[…] in addition to allowing me to donate milk” (M33);
Positive unspecified experiences	“*Very important” (M2);* “great” (M22); “Very good” (M26); “Great! Essential!” (M31); “It was great.” (M34); “Very good” (M47);
**Difficulties encountered**	
Distance from rooms	“[…]The only difficulty is that in the branch I worked […] the breastfeeding room was a 10-min walk from the place where I worked, adding up the time pumping, I ended up staying too long in there, plus the time to return to my workplace. Even though I was a manager, I was still embarrassed.” (M11);
Need to stop using the rooms due to the age of the child.	“Using the room was great, but due to company policy, I only had the right to be off work in the first month […]. I came back from maternity leave, plus vacation time, and my daughter was 7 months old, so by 8 months, I was forced to introduce formula (Suggestion: we should be free to use the room for as long as necessary for other activities… such as for example, a medical examination to ensure that breastfeeding resumes.” (M49).

However, we identified some difficulties regarding room usage, although they were not indicated as negative by women, such as: (1) distance from the rooms to the workplace, and (2) need to stop using the rooms due to the child's age (Table 3).

### Women's Perception of BSR

The female participants had positive opinions about the BSRs about (1) the importance of the rooms for different purposes, (2) the respect of working women, and (3) the atmosphere (physical structure and equipment). On the other hand, the negative perceptions are related to (1) little use, (2) the small amount of BSRs implemented by companies, and (3) the need to reinforce this action. The thematic analysis is available in Table 4.

**Table 4:** Perceptions of female employees about breastfeeding support rooms (*n* = 53), Brazil, 2019–2020.

**Perceptions**	**Quotes**
**Positive**	
Importance of BSRs	“I consider it extremely important with immeasurable repercussions […]” (M3); “extremely essential” (M4); “It is indispensable […]” (M8); “They are essential” (M11); “fundamental for those who want to breastfeed” (M18); “There's nothing better, super necessary” (M23); Necessary. Indispensable […] improves the quality of life of the whole family because if the mother is well, the family will also be” (M26);
Importance of BSRs for women	“Essential for working mothers who are aware of the importance of breast milk.” […] (M1); “Necessary for mothers who breastfeed, regardless of how long.” (M10); “A space necessary so that mothers do not have to be exposed or go through constraints when feeding their children. […] (M17); “It is fundamental to our process […] (M20) “It is a fundamental support for mothers who want and/or must breastfeed” (M29); “A standout for us mothers” (M32);
Importance of BSRs for the child	“[…] These rooms are essential for the babies' development […] (M1); “[…] the Ministry of Health proves that breastfeeding contributes to the child's health” (M3); “It is beneficial for mothers and especially for babies who will continue to be breastfed longer” (M24);
Importance of BSRs for breastfeeding	“Essential to encourage exclusive breastfeeding” (M2); […] Makes a lot of difference in support for breastfeeding” (M12); “encourages breastfeeding and that women breastfeed longer” (M19); “Helps in the process of continuity of breastfeeding and donation of breast milk” (M21); “[…] is essential for the continuity of breastfeeding […] (M41); “[…] a unique space that contributes to increasing breastfeeding time” (M47);
Importance of BSRs for job performance	“[…] And a satisfied employee with her needs well-met is more productive at work” (M27); “[…] and also for the comfortable performance of their job activities” (M41);
Appreciation of the woman employee	“[…] it is a way for the company to demonstrate that it cares about women and to promote the well-being and health of mothers and children” (M3); “It makes a difference for women who live in conflict between their family l and work life. The company that has one is saying: We support you at this moment, everything is fine” (M7); “Represents the appreciation of the working woman, providing due support in such a difficult moment of returning from family leave and being apart from the baby” (M16); “[…] It demonstrates respect, attention, and care for their employees” (M17); “It is sensational, having an exclusive place for moms to take fresh milk to their child […] five-star company” (M22);
Ambiance	“Excellent, it has everything necessary” (M5); “Very cozy and quiet room” (M9); “It is comfortable, safe, hygienic and private for the mother” (M30); “The environment makes us well at ease, it is clean and cozy, with a fridge and freezer, hot water in the sink, alcohol, and tissue” (M43); “the environment is very good, clean, calm, totally adequate” (M44); “It is a reserved, clean place, with the necessary appliances (refrigerator, couch, sink, etc.) that makes the woman feel at ease” (M46);
**Negative**	
Little use	“*It's super interesting. But in practice, it only worked for me […]” (M14); “[…] but since the room administrator had left the company, the place was a little neglected,one of the faucets didn't work, for example, and it was not cleaned often. But there was a refrigerator, a couch and a key. With this, I realized that few mothers used the room, I think it is a cultural issue and also more difficult for the mothers of the operational team” (M45);*
Little existence	”*There is almost no such space” (M48);*
Need for reinforcement	“*[…] I believe that the importance is even greater for companies that grant only 4 months of maternity leave” (M1); “all companies should have […] (M12); “indispensable, every company should have” (M13); “I think it should remain and be further developed. With clear policies regarding use that aim to help mothers and families maintain breastfeeding” (M49); “all companies should have one to encourage mothers to continue breastfeeding after returning to work” (M42)*

### Relationship Between the Use and Perceptions About the BSRs and the 2030 Goals to Increase Breastfeeding Rates Worldwide and Achieve SDG

From the codings and themes resulting from the experiences using the BSRs and the opinions of women who use BSRs, we were able to establish the positive relationship between the BSRs and the international goals to raise breastfeeding indicators worldwide, and to several of the SDG.

We observe a strong relationship between BSRs and SDG 8, particularly regarding decent work and economic growth, as breastfeeding in the workplace can promote decent work, women's productivity and contribute to economic growth.

In Table 3, some reports show that BSRs provide comfort, physical, and emotional well-being for women to perform their activities, with comments such as “It was wonderful, I felt welcomed, safe, cared for, very calm” (M23); “[…] A major benefit was being able to pump milk and avoid mastitis, which happened when I had my first daughter, precisely because I had no way to pump milk.” (M15); and “[…] The room was essential. […] Also, to relieve any discomfort throughout the day.” (M29). In Table 4, we can see how the BSR contributes to a good relationship between employees and employers. Once women recognize the importance of the BSRs for the full realization of their professional activities, they realize that BSRs contribute to the appreciation of the women in the workforce since they feel happy and valued professionally: “[…] And a satisfied employee with her needs well-met is more productive at work” (M27) and “[…] and also for performing their job activities smoothly” (M41).

In addition, the discourse surrounding women points to an appreciation of the differential that the company provides for them. Women recognize and value a company that meets their needs as a woman, as a mother, and as a professional: “[…] it is a way for the company to demonstrate that it cares about women and to promote the well-being and health of mothers and children” (M3), and “It makes a difference for women who live in a conflict between their family and work life. The company has a saying: We support you at this moment, everything is fine” (M7).

We observed a potential relationship between BSR availability in work environments and the achievement of SDG 5 concerning gender equality. As observed in Tables 3, 4, BSRs contribute to women having the opportunity to perform their professional activities just as well as men, without physical or emotional stress, feeling valued and recognized professionally.

We can also see indirect relationships between BSRs and the achievement of six other SDGs from the increase in breastfeeding rates, which positively impact the achievement of the following SDG: (1) end poverty (SDG 1): breastfeeding contributes to higher financial income for breastfed adults, (2) zero hunger (SDG 2): breastfeeding reduces hunger and malnutrition, promoting total child growth, and development in the early years of life, (3) good health and well-being (SDG 3): breastfeeding improves the physical and mental health, and well-being of mothers and children, (4) quality education (SDG 4): breastfeeding contributes to an increase in intelligence quotient. Breastfed children for longer participate in more educational activities, (5) reducing inequalities (SDG 10): breastfeeding provides the best start in life. With the increase in breastfeeding rates, there is a decrease in infant morbidity and mortality, an increase in physical and emotional well-being, in income when adult, thus reducing social inequalities throughout generations, (6) climate action (SDG 13): breastfeeding is sustainable and does not harm the environment, unlike baby formulas that can contribute to the greenhouse effect and waste generation, and should only be used when necessary. This relationship can be seen in Table 5.

**Table 5:** Relationship between Breastfeeding Support Rooms and their contribution to achieving Sustainable Development Goals, Brazil, 2019–2020.

		**Relationship with the SDG**		**SDG directly and indirectly affected by BSRs**
	→	BSRs have a strong relationship with SDG	→	SDG 8: Promote sustained, inclusive, and sustainable economic growth, full and productive employment, and decent work for all
				SDG 5: Achieve gender equality and empower all women and girls
Breastfeeding Support Rooms	→	BSRs contribute to raising the rates of breastfeeding, which has a potential relationship with the SDG	→	SDG 1: End poverty in all its forms everywhere
				SDG 2: End hunger, achieve food security and improved nutrition and promote sustainable agriculture
				SDG 3: Ensure healthy lives and promote well-being for all of all ages
				SDG 4: Ensure inclusive and equitable quality education and promote lifelong learning opportunities for all
				SDG 10: Reduce inequality within and among countries
				SDG 13: Take urgent action to combat climate change and its impacts

*The implementation of policies to promote, protect and support breastfeeding has the potential to reinforce breastfeeding in general, including breastfeeding in the work environment and BSR, in addition to contributing to SDG 17 on global partnerships and joint action between governments and civil society*.

## Discussion

From the reports of formal female workers, we identified how their experiences using BSRs, their perceptions and opinions on the BSR strategy, and the support for breastfeeding at work as a result of implementing BSRs, can contribute directly to SDG 8 and SDG 5 and consequently lead to six other SDGs as the time women continue breastfeeding increases. Although there are studies that investigate BSRs, we could not find studies with this focus. Our sample corresponded to a little more than 40% of active BSR users in the state of Paraná, which we consider an acceptable standard for studies with self-completion questionnaires.

Most women were middle-aged, between 31 and 40 years old, married or living in a common-law marriage. Regarding the positions occupied by women at work, we observed that most were placed in administrative functions. According to Dagher et al. (7), women who work in an office have a higher chance of initiating breastfeeding. In addition, research shows that the sociodemographic conditions of women in southern Brazil are favorable for exclusive breastfeeding but that it is a challenge to keep breastfeeding for more than 12 months (20).

Most of the women in our study had graduated college, worked up to 8 h a day, and were encouraged by the company and their superiors to take breaks to pump breast milk. These findings corroborate with Tsai (21) in a study conducted in Taiwan with 715 female employees.

We know that the high level of education can be a favorable factor for breastfeeding [Tsai (20, 21)], thus contributing to women having more positive perceptions about the actions that contribute to the maintenance of breastfeeding, including the BSRs. We emphasize, however, that only 6 (11.3%) of the women held higher management positions, and it was unanimous for all users, even female workers, and assistants, to be satisfied with being able to use SAA in the workplace. Furthermore, in our study, 100% of the women had a late return to work after the 180-day maternity leave, and it is common for workers to extend this period with 30 vacation days, resulting in a return to work of around 7 months after the baby is already on complementary feeding. In addition, most women also had the privilege of using BSRs even after the time established in the labor legislation of breaks up to 6 months. We believe that this was due to employers becoming more aware of the importance of breastfeeding.

The Citizen Company (Empresa Cidadã) program, instituted in 2008 by Law 11.770 (22), is aimed at the optional extension of maternity leave from 120 to 180 days through the concession of a tax incentive. The extension of maternity leave is an achievement that has ensured progress for public policies in support of early childhood in Brazil. Six-month maternity leave is associated with an increased prevalence of exclusive breastfeeding (8). All the companies participating in this study have adopted a 180-day maternity leave, and most have also adopted a 20-day paternity leave.

Most of the women had worked for a long time in the company. From their accounts, as seen in Table 4, we found that employees perceive that the company, upon implementing BSRs, values their work and provides conditions for a good work performance, contributing strongly to the achievement of SDG 8 and 5. This relationship between BSRs and SDG 8 and 5 was more specifically about promoting decent work, allowing women to find physical and emotional comfort that enables them to be more relaxed, and willing to perform their professional activities on equal grounds.

We noticed that the BSRs contribute to the appreciation of female employees. For example, women reported feeling happy and valued professionally for having rooms available since they feel that the company supports them in their needs. In addition, we found that female employees recognize the company's advantage, which is related to an increase in satisfaction and contentment concerning the company they work for, as observed in some reports in Table 4.

This finding corroborates the assertion of Rollins et al. (3) that BSRs can reduce absenteeism, improve work performance, engagement, and workforce retention. We understand that BSRs have a great potential to reduce barriers to continued breastfeeding among working women, promote decent work, which concerns SDG 8, and are a low-cost investment that employers could easily implement.

By allowing women to alleviate their physical discomfort during the workday and find emotional well-being and comfort, the relationship between BSRs and gender equality merges with the relationship between BSRs and decent work. When women can use the BSRs, the environment is considered safe and comfortable. In addition, they have emotional comfort because the woman knows that her child will safely drink her milk stored in an appropriate place during her professional activities and avoid using baby formula. Therefore, women can dedicate themselves to their professional activities more efficiently, allowing for equal working conditions as men, who do not have such concerns. However, to the best of our knowledge, no studies support our findings regarding how BSRs could contribute to gender equality related to SDG 5, seeing as this field is still little explored. Therefore, we highlight the need for further studies on this topic.

In Tables 3, 4, we can see that women relate the importance of the SAA with the maintenance and continuity of breastfeeding. Moreover, several authors corroborate the relationship between BSRs and increased breastfeeding rates (3, 12–16).

We consider that increasing breastfeeding rates has the potential to positively impact at least six of the SDGs, like Victora et al. (2), who relate the promotion of breastfeeding to several SDGs.

There is a relationship between breastfeeding and reducing poverty and increasing human capital, which, in turn, can also reduce social inequalities. According to Victora et al. (23), breastfeeding improves human capital and significantly affects adult education and income. Breastfed children have better intellectual development for a more extended period (average increase of 3 IQ points). As a result, they can show positive results in school performance and a higher income in the long term. These findings relate to SDGs 1, 4, and 10.

The relationship between breastfeeding, maternal, and child health, included in SDG 3, is supported by several authors. Breastfeeding benefits both children's (2, 3, 23, 24) and women's health (2, 3, 25) and strengthens the emotional bond between the mother and baby. Breastfeeding reduces infant deaths (2, 6, 24, 26, 27) and can prevent 13% of worldwide deaths in children under 5 years of age (6, 26). Extending breastfeeding to an almost universal level could prevent 823,000 annual deaths of children under 5 and 20,000 annual deaths from breast cancer (2).

Breastfeeding ensures food and nutritional security and promotes total growth and development for children. Breastfeeding is a child's first protection against hunger, malnutrition, illnesses, and is also their most lasting investment in physical, cognitive, and social aspects (24) related to SDG 2.

According to the women, BSRs are essential because they can avoid using baby formulas, as seen in Tables 3, 4. This fact can be related to the achievement of SDG 13, which concerns reducing the impacts of climate change. In addition, breastfeeding is sustainable and does not harm the environment, unlike baby formulas, which contribute to the greenhouse effect and waste generation, and thus, should only be used when necessary. According to Rollins et al. (3), breastfeeding has economic and environmental benefits.

Managers have a critical role in the success of breastfeeding for working women (28). We know that lack of breastfeeding support in the workplace and inconvenient breastfeeding conditions discourage women from maintaining the practice (6, 21, 29, 30).

In our study, most women reported that they receive support and encouragement from their companies. However, from the experience of two BSRs users, one of the barriers when using the rooms is related to the fact that they would have liked to have used the BSRs longer. One of these participants was discouraged from using the BSR after her child turned 8 months. This fact indicates that despite the government's efforts to encourage BSRs, this strategy can be improved.

Through Convention 183, the ILO proposes measures to protect maternity and breastfeeding in the workplace, assigning countries the responsibility to institute a period for breastfeeding breaks, the reduction of daily working hours, the duration of breastfeeding breaks, among others, as noted in items 1 and 2 of article 10 (31).

According to Rollins et al. (3), 130 countries have paid breastfeeding breaks, seven countries have unpaid breaks, and 45 countries do not have a policy for breastfeeding at work. In Brazil, the right to a breastfeeding break date back to the 1940s and is included in the labor legislation. Article 396 of the Consolidation of Labor Laws (CLT) determines that a woman should have two 30-min breastfeeding breaks during the workday until the child is 6 months old. The law also incorporates an extension to the 6 months at the discretion of competent authorities, depending on the child's health (32).

These results reinforce the importance of an update to Brazilian labor laws concerning an extension of breaks to breastfeed or pump milk during the workday, either if breastfeeding continues or at least until the child is 2 years old, which is the recommended threshold for breastfeeding.

This right to breastfeed children up to 2 years of age must be established by law and available to all women, regardless of the manager's goodwill. The institutionalization of the right to breastfeed at work for the first 2 years of the child's life would also contribute to achieving the international breastfeeding goals for 2030, which are 80% breastfeeding by age one and 60% by age two (1). To that end, governments, donors, and civilians must commit and invest in the promotion, protection, and support of breastfeeding (3).

There are some limitations to our study that need to be pointed out. First, we focused only on one state in Brazil, which has 76.2% of SAA in the southern region of the country and 8% of the total 200 BSRs certified in Brazil by 2017. Brazil is a very diverse country; thus, it would be beneficial to use complementary studies to verify our findings in other regions. Second, our study involved companies that adhered to other support strategies for working women, such as extending maternity and paternity leave, which may have influenced the positive perception of women about the Breastfeeding Support Rooms. Finally, the use of the online questionnaire instead of in-depth interviews may have made it challenging to capture women's perceptions somehow.

However, despite these limitations, this is the first study in Brazil to gather data on women's experience using BSRs and their perception about it. This is also the first study that points to the great potential relationship between BSRs and the achieving SDG 5 and 8 by promoting decent work, professional appreciation, comfort, and physical and emotional well-being, all factors that contribute to work productivity and economic growth besides impacting other SDGs by increasing breastfeeding rates.

Our findings conclude that the BSR is seen as an important and cheap strategy for female employees. Therefore, countries should invest to promote breastfeeding in the workplace through BSRs because they contribute directly and indirectly to achieving the SDG.

## Data Availability Statement

The raw data supporting the conclusions of this article are in Portuguese and will be provided by the authors, without reservation, upon a request sent *via* email to the corresponding author.

## Ethics Statement

The studies involving human participants were reviewed and approved by the Ethics Committee of the Health Sciences Department of the Federal University of Paraná, under Certificate of Presentation of Ethical Appreciation (CAAE) number 65401917.5.3004.5225. The patients/participants provided their written informed consent to participate in this study.

## Author Contributions

CBS collected, analysed, and interpreted the data and drafted and reviewed the manuscript. SIV and RPGVCS interpreted the data and critically reviewed the manuscript. All authors contributed to the conception and design of the study and approved the final version to be published.

## Conflict of Interest

The authors declare that the research was conducted in the absence of any commercial or financial relationships that could be construed as a potential conflict of interest.

## Publisher's Note

All claims expressed in this article are solely those of the authors and do not necessarily represent those of their affiliated organizations, or those of the publisher, the editors and the reviewers. Any product that may be evaluated in this article, or claim that may be made by its manufacturer, is not guaranteed or endorsed by the publisher.
